# Radiomics Analysis on Gadoxetate Disodium-Enhanced MRI Predicts Response to Transarterial Embolization in Patients with HCC

**DOI:** 10.3390/diagnostics12061308

**Published:** 2022-05-24

**Authors:** Roberto Cannella, Carla Cammà, Francesco Matteini, Ciro Celsa, Paolo Giuffrida, Marco Enea, Albert Comelli, Alessandro Stefano, Calogero Cammà, Massimo Midiri, Roberto Lagalla, Giuseppe Brancatelli, Federica Vernuccio

**Affiliations:** 1 Section of Radiology, Department of Biomedicine, Neuroscience and Advanced Diagnostics (BiND), University Hospital “Paolo Giaccone”, Via del Vespro 129, 90127 Palermo, Italy; dott.francescomatteini@gmail.com (F.M.); massimo.midiri@unipa.it (M.M.); roberto.lagalla@unipa.it (R.L.); gbranca@yahoo.com (G.B.); 2 Department of Health Promotion, Mother and Child Care, Internal Medicine and Medical Specialties, PROMISE, University of Palermo, 90127 Palermo, Italy; celsaciro@gmail.com (C.C.); paologiuffrida1@gmail.com (P.G.); marco.enea@unipa.it (M.E.); calogero.camma@unipa.it (C.C.); 3 University of Palermo, Piazza Marina, 61, 90133 Palermo, Italy; carlacamma@gmail.com; 4 Department of Surgical, Oncological and Oral Sciences (D.C.O.S.), University of Palermo, 90133 Palermo, Italy; 5 Ri.MED Foundation, Via Bandiera 11, 90133 Palermo, Italy; acomelli@fondazionerimed.com; 6 Institute of Molecular Bioimaging and Physiology, National Research Council (IBFM-CNR), Contrada Pietrapollastra-Pisciotto, 90015 Cefalù, Italy; alessandro.stefano@ibfm.cnr.it; 7 Department of Radiology, University Hospital of Padova, Via Nicolò Giustiniani, 2, 35128 Padua, Italy

**Keywords:** radiomics, LI-RADS, hepatocellular carcinoma, magnetic resonance imaging, treatment response

## Abstract

Objectives: To explore the potential of radiomics on gadoxetate disodium-enhanced MRI for predicting hepatocellular carcinoma (HCC) response after transarterial embolization (TAE). Methods: This retrospective study included cirrhotic patients treated with TAE for unifocal HCC naïve to treatments. Each patient underwent gadoxetate disodium-enhanced MRI. Radiomics analysis was performed by segmenting the lesions on portal venous (PVP), 3-min transitional, and 20-min hepatobiliary (HBP) phases. Clinical data, laboratory variables, and qualitative features based on LI-RADSv2018 were assessed. Reference standard was based on mRECIST response criteria. Two different radiomics models were constructed, a statistical model based on logistic regression with elastic net penalty (model 1) and a computational model based on a hybrid descriptive-inferential feature extraction method (model 2). Areas under the ROC curves (AUC) were calculated. Results: The final population included 51 patients with HCC (median size 20 mm). Complete and objective responses were obtained in 14 (27.4%) and 29 (56.9%) patients, respectively. Model 1 showed the highest performance on PVP for predicting objective response with an AUC of 0.733, sensitivity of 100%, and specificity of 40.0% in the test set. Model 2 demonstrated similar performances on PVP and HBP for predicting objective response, with an AUC of 0.791, sensitivity of 71.3%, specificity of 61.7% on PVP, and AUC of 0.790, sensitivity of 58.8%, and specificity of 90.1% on HBP. Conclusions: Radiomics models based on gadoxetate disodium-enhanced MRI can achieve good performance for predicting response of HCCs treated with TAE.

## 1. Introduction

Hepatocellular carcinoma (HCC) accounts for about 90% of all primary liver malignancies in cirrhotic patients [1]. Treatment options for HCC are based on tumor extent and severity of chronic liver disease [1]. In patients with localized HCC, ineligible for resection or transplantation and without macrovascular invasion, locoregional treatments represent an important therapy option to prolong patients’ survival. Among these, intraarterial treatments, such as transarterial chemoembolization (TACE) and transarterial embolization (TAE), are widely used as first-line treatments in patients with intermediate stage (Barcelona Clinic Liver Cancer stage B) HCC, not eligible to curative surgical resection, or as a bridge to transplantation [1,2]. Accurate prediction of response to intraarterial treatments is crucial for optimal patients’ selection. Response to treatment can be heterogeneous according to pre-treatment tumor characteristics. Several prior clinical models have been proposed for predicting treatment response with varying results, including assessment of clinical and tumor features such as location and size of HCC, number of nodules, α-fetoprotein, patients age, liver function, and performance status [3,4]. Additionally, some studies investigated pretreatment qualitative imaging features of HCC on magnetic resonance imaging (MRI) for predicting tumor response [5,6]. Gadoxetate disodium-enhanced MRI has an important role for the diagnosis and pretreatment assessment in patients with HCC, providing improved sensitivity for the diagnosis and high quality of the hepatobiliary phase (HBP). However, qualitative imaging features may be affected by subjective interpretation, readers experience, and different definitions among HCC guidelines. Therefore, reliable prediction of response on pretreatment MRI remains an unsolved challenge in clinical practice.

Radiomics is emerging as a promising tool that allows to extract a large number of quantitative features from radiological images. Radiomics features can be combined with clinical and imaging data for building predictive models for lesion characterization, prediction of treatment response, and patients’ prognosis [7,8]. Initial studies explored the potential of radiomics and texture analysis for the prediction of treatment response and prognosis after intraarterial treatments on contrast-enhanced CT [9,10,11,12,13,14,15,16] and MRI with extracellular contrast agents [17,18,19,20,21,22]. On MRI, radiomics analysis was applied to T1-weighted post-contrast images, T2-weighted sequences, and diffusion weighted imaging with high performances [17,18,19,20,21,22]. However, none of these studies investigated accuracy of radiomics on gadoxetate disodium-enhanced MRI in combination with both clinical and semantic imaging features. Moreover, the impact of different model developments on the final diagnostic performance has not been compared in this setting. We hypothesize that radiomics may provide an added value for predicting treatment response of HCC by quantifying lesion heterogeneity related to tumor aggressiveness that cannot be appreciated by the radiologist eyes. 

The aim of this study was to explore the potential of radiomics on gadoxetate disodium-enhanced MRI in comparison with clinical variables and qualitative imaging features for predicting hepatocellular carcinoma response after transarterial embolization, using statistical and computational models.

## 2. Materials and Methods

This retrospective, single-institution study protocol was approved by the Ethical Committee of University Hospital of Palermo with a waiver for informed consent (N. 10/2020 Approval Date: 25 November 2020). 

### 2.1. Population

**Figure 1:**
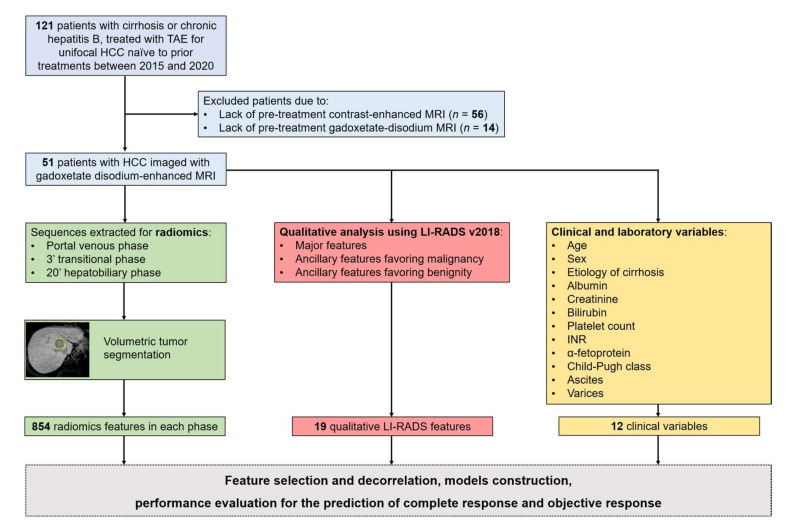
Flowchart of patients’ accrual for the study. Abbreviations: HCC: Hepatocellular Carcinoma; LI-RADS: Liver Imaging Reporting and Data System; MRI: Magnetic Resonance Imaging; TAE: Transarterial Embolization.

A search through the clinical and radiological database at our Institution was conducted to select adult patients meeting the following inclusion criteria: (1) diagnosis of cirrhosis or chronic hepatitis B infection; (2) presence of unifocal HCC naïve to any prior treatment, in patients not candidate to surgical resection due to advanced liver disease; (3) gadoxetate disodium-enhanced MRI performed at our Institution; (4) underwent successful transarterial embolization between 2015 and 2020, within one month of the pretreatment gadoxetate disodium-enhanced MRI. Patients were excluded if they met any of the following criteria: (1) lack of pretreatment contrast-enhanced MRI exams (*n* = 56); (2) pretreatment contrast-enhanced MRI not acquired with gadoxetate disodium as contrast agent (*n* = 14). The flowchart of patients’ accrual for this study is illustrated in Figure 1.

The final study population consisted of 51 patients (37 males, 14 females, median age 73 years, range 44–85 years) with unifocal HCC and available pretreatment gadoxetate disodium-enhanced MRI. Patient-related clinical data and laboratory variables—i.e., age at the time of treatment, sex, laboratory and tumor markers, history of ascites or varices, and Child-Pugh score—were collected using the electronic data repository systems.

### 2.2. MRI Technique

MRI exams were acquired on two clinical 1.5-T MR scanners (*n* = 27 patients with Signa Excite, General Electric, Healthcare, Milwaukee, WI, USA; and *n* = 24 patients with Intera Achieva 1.5 Philips Healthcare, Best, The Netherlands) equipped with a 16-channel body phased-array coil. All patients underwent contrast-enhanced MRI with a dedicated liver protocol, in accordance with LI-RADS v2018 technical recommendations [23] which include the following sequences: axial T2-weighted turbo or fast spin-echo (with and without fat saturation) sequences, axial dual gradient-recalled echo T1-weighted sequence (in-phase and opposed-phase), and axial diffusion weighted imaging acquired with b values of 0, 150 and 800 s/mm^2^. Axial T1-weighted three-dimensional gradient-recalled echo sequences with fat suppression (Liver Acquisition with Volume Acceleration, LAVA, General Electric; or T1-weighted high-resolution isotropic volume examination, THRIVE, Philips Healthcare) were obtained before and after contrast agent administration. Detailed parameters of T1-weighted three-dimensional gradient-recalled echo sequences are reported in Supplementary Table S1. 

A weight-based dose of 0.025 mmol/Kg of gadoxetate disodium (Gd-EOB-DTPA, Primovist, Bayer Healthcare, Berlin, Germany) was injected at 1 mL/sec, followed by 20-mL saline flush at the same injection rate, using an automatic injector (Medrad^®^ Spectris Solaris^®^ EP, Bayer Healthcare, Berlin, Germany). Post-contrast phases were acquired on late hepatic arterial (12–14 s after the detection of contrast bolus), portal venous (PVP, 50–60 s after the detection of contrast bolus), transitional (at 3 min and 5 min), and hepatobiliary (HBP, 20 min) phases.

### 2.3. MRI Qualitative Analysis

Two radiologists (F.V. and R.C., with seven and six years of experience in liver imaging), blinded to the lesions’ outcome, reviewed all the gadoxetate disodium-enhanced MRI exams in consensus using the LI-RADS v2018 diagnostic algorithm [24]. The radiologists recorded the presence of major features including size, nonrim arterial phase hyperenhancement (nonrim APHE), nonperipheral “washout” (evaluated on PVP only due to the injection of gadoxetate disodium), enhancing “capsule”, threshold growth, and assigned a final category based on LI-RADS diagnostic table [24]. Additionally, ancillary features favoring malignancy (not HCC in particular or HCC in particular), and ancillary features favoring benignity were recorded as defined by LI-RADS algorithm [24]. Notably, due to the very low frequency of ancillary features favoring benignity in our cohort (only HBP isointensity detected in one observation) they were not included for further analysis. 

### 2.4. Segmentation and Radiomics Feature Extraction

**Figure 2:**
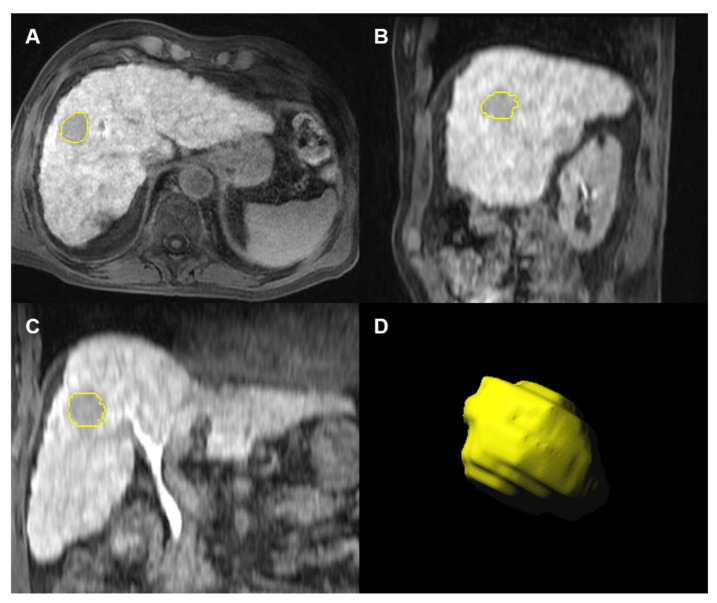
Gadoxetate disodium-enhanced MRI before transarterial embolization in a 74-year-old man with hepatitis C-related cirrhosis and a 27-mm hepatocellular carcinoma. Images shows example of whole tumor segmentation (circles) on axial (**A**), sagittal (**B**), and coronal (**C**) hepatobiliary phase images with volumetric lesion reconstruction (**D**).

Lesion segmentation was performed using the research software Radiomics, version 1.2.2 (Siemens Healthineers, Forchheim, Germany), by the same radiologists in consensus, with an interval time of one month from qualitative analysis to avoid recall biases. Post-contrast axial T1-weighted three-dimensional gradient-recalled echo sequences acquired on PVP, 3′ transitional, and HBP were used for lesion segmentation and radiomics feature extraction. T2-weighed and diffusion weighted imaging were already assessed in prior investigations [19,20]. The late hepatic arterial phase was not included due to the known possible respiratory-motion artifacts in the images acquired after the injection of gadoxetate disodium compared to other gadolinium-based contrast agents, and to limit the confounding factors associated with the contrast injection rate [25,26]. Selected sequences were anonymized and sent to a dedicated workstation equipped with a research radiomics software (Radiomics, version 1.2.2; Siemens Healthineers, Forchheim, Germany) [27]. A region of interest was manually drawn within the lesion margins, including the whole tumor visualized in consecutive slices (Figure 2). The radiomic tool accesses the PyRadiomics package (Version 1.3.0) implemented in Python which calculates 854 radiomics features categorized into three main categories (i.e., intensity, shape, and texture radiomics features), including 18 first order histogram-based features, 24 gray-level co-occurrence matrix (GLCM), 14 gray level dependence matrix (GLDM), 16 grey-level run-length matrix (GLRLM), 16 grey-level zone length matrix (GLZLM), 5 neighboring gray tone difference matrix (NGTDM), 17 shape-based, and 743 wavelet-based radiomics features. 

A detailed description of the features can be found in the online documentation of PyRadiomics (https://pyradiomics.readthedocs.io/en/latest/features.html, accessed on 20 January 2022).

### 2.5. Lesions Outcome 

Evaluation of treatment response at contrast-enhanced exams performed at one month after treatment was used as reference standard for this study. Treatment response was evaluated using the modified RECIST criteria (mRECIST) [28]. Patients were classified as complete response (disappearance of any intratumoral arterial enhancement), partial response (≥30% decrease of intratumoral enhancement of target lesion), stable disease (neither partial response or progression disease), and progressive disease (≥20% increase in size of target lesion) [28]. Patients with complete response and partial response were considered as objective response.

### 2.6. Statistical Analyses of Qualitative Features

Categorical variables were expressed as numbers and percentages. Continuous variables were reported using median and interquartile ranges (IQR) according to the Shapiro–Wilk normality test. Analysis of clinical variables and LI-RADS qualitative imaging features was conducted using the Pearson χ^2^ or Fisher exact test to assess differences between categorical variables, and Mann–Whitney U test to assess differences between continuous variables. A *p* value < 0.05 was considered for statistical significance. Statistical analyses were performed using SPSS Software (Version 20.0. Armonk, NY, USA: IBM Corp.).

### 2.7. Radiomics Models Construction

Two different radiomics models were built, including a statistical model based on logistic regression with elastic net penalty (model 1) and a computational model based on an innovative hybrid descriptive-inferential feature extraction method that combines the point biserial correlation and the logistic regression (model 2) [29,30]. Each model was tested for the prediction of complete response and objective response in the analyzed phases (PVP, 3′ transitional, and HBP). The models were constructed including the dataset of 854 radiomics features, the combined dataset of 12 clinical variables, and 19 LI-RADS qualitative imaging features. Predictive performances of the models were summarized by using receiver operating characteristics (ROC), areas under the ROC curve (AUC) with 95% confidence interval (95% CI), sensitivity, specificity, and accuracy.

### 2.8. Radiomics Model 1

For the logistic regression model, the dataset on each phase was randomly split into training set (80%) and test set (20%). For features selection, a logistic model with elastic net (e-net) penalty was fit on the training set. The tuning hyper-parameters alpha and lambda were found in the following way: a grid search of alpha values is carried out in the interval [0.5, 1] and, for each fixed alpha, the best lambda is found by a 5-fold cross-validation. The e-net is preferred to the LASSO in order to reduce the chance of selecting zero features. The interval [0.5, 1] is chosen as in this step we are more interested in feature selection than decorrelating the features.

For features decorrelation, two reduced training set and test set were created on the ground of the selected features. A logistic regression model with ridge penalty [31] was fit, while lambda is newly estimated by 5-fold cross-validation. 

Statistical analyses were performed by a dedicated statistician (M.E. with 16 years of experience) by using the R packages glmnet [32] and islasso [33] at steps 1–2, respectively. 

### 2.9. Radiomics Model 2

For the computational model, the point-biserial correlation coefficient (rpb) was calculated between each feature and the corresponding outcome in order to obtain the most discriminative features, avoiding overfitting problems and repetition of multiple features with high similarity or with incidental statistical significance [29,30]. The absolute value of rpb was used to sort the radiomics features in descending order. Next, a cycle was started to add one column at a time performing a logistic regression analysis. The *p* value of the current iteration was compared with the previous iteration. If this did not decrease, the cycle was interrupted. In this way, the features with valuable association with the outcome were identified. Subsequently, the Discriminant Analysis (DA) was used as a method for features classification [34] in order to overcome the unbalanced dataset issue which occurred in the case of complete response where 14 patients were compared with 37 patients (see Section 3.2). As reported in prior studies [35,36]. unbalanced datasets do not have a negative effect on DA performance. Furthermore, Model 2 was tested in a k-fold cross-validation fashion grouping data into training and test sets maintaining the same outcome percentage of the original dataset to preserve any data imbalances. In addition, the “k-fold” strategy was used in such a way as to guarantee disjointed test sets. Thus, the dataset was divided into equal k = 5 subsets, and the holdout method was repeated 5 times. In other words, each time a different fold never used during the training was left for test. Then, the performance of each k-model was averaged to make the results more robust, and to avoid optimistic results. The k = 5 was empirically determined through the trial-and-error method (k range: 5–15, step size of 5).

Computational analysis of radiomics features was conducted by a computer engineer (A.C., with 9 years of experience on computational data analysis).

## 3. Results

### 3.1. Population

Overall, 51 unifocal treatment-naïve HCCs (median size 20 mm, IQR 16–30 mm) imaged with gadoxetate disodium-enhanced MRI were analyzed in the final population. Forty-seven (92.2%) lesions were classified as LR-5, while four (7.8%) were categorized as LR-4, and proved to be HCC at percutaneous liver biopsy. According to mRECIST criteria, 14 (27.4%) patients were classified as complete response, 15 (29.4%) as partial response, 19 (37.3%) as stable disease, and 3 (5.9%) as progressive disease. Objective response (complete and partial responses) was obtained in 29 (56.9%) patients. 

### 3.2. Clinical and Qualitative Imaging Analysis

Differences in clinical and laboratory variables according to the response assessment are reported in Table 1. Only platelet count resulted significantly higher in patients with complete response compared to patients lacking complete response (median 115.1 × 10^3^/μL vs. 80.0 × 10^3^/μL; *p* = 0.021). No statistically significant differences were observed in patients with or without objective response. 

There were no statistically significant differences in frequency of LI-RADS major features and ancillary features favoring malignancy in HCCs according to treatment response (Table 2). 

### 3.3. Performance of Radiomics-Based Models

#### 3.3.1. Radiomics Model 1

The most discriminative features selected from clinical variables, LI-RADS qualitative imaging features, and radiomics-based features through the logistic regression with elastic net penalty are reported in Supplementary Table S2.

**Figure 3:**
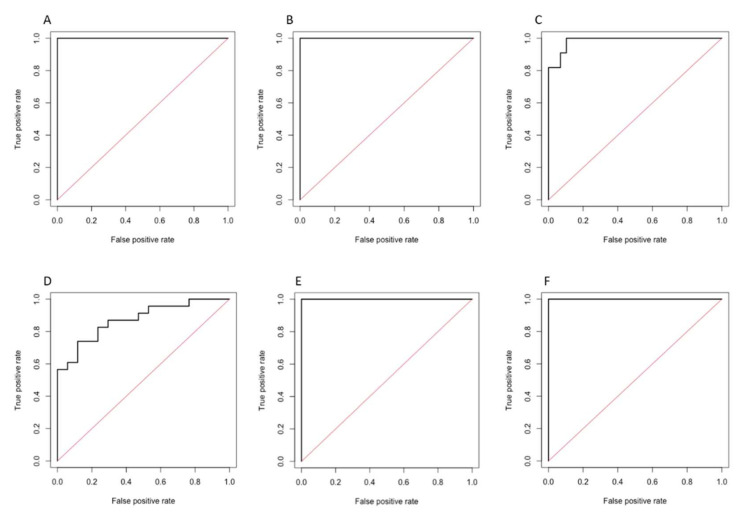
ROC curves of the radiomics model 1 in the training set for predicting complete response on portal-venous (**A**), 3′ transitional (**B**), and hepatobiliary (**C**) phases, and for predicting objective response on portal-venous (**D**), 3′ transitional (**E**), and hepatobiliary (**F**) phases.

**Figure 4:**
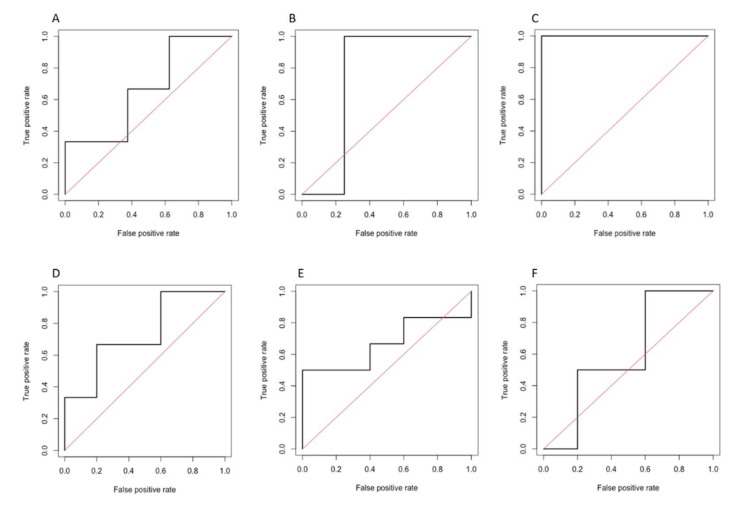
ROC curves of the radiomics model 1 in the test set for predicting complete response on portal-venous (**A**), 3′ transitional (**B**), and hepatobiliary (**C**) phases, and for predicting objective response on portal venous (**D**), 3′ transitional (**E**), and hepatobiliary (**F**) phases.

Performances of the statistical models tested by using the logistic regression are reported in Table 3 for the training set, and Table 4 for the test set. Corresponding ROC curves are illustrated in Figure 3 for the training set, and in Figure 4 for the test set. Model 1 on HBP showed the highest performance in the test set for predicting complete response, with an AUC of 1.000 (95% CI 1.000–1.000, *p* < 0.001), sensitivity of 100%, and specificity of 100%. Model 1 on PVP showed the highest performance in the test set for predicting objective response, with an AUC of 0.733 (95% CI 0.405–1.000, *p* = 0.163), sensitivity of 100%, and specificity of 40.0%.

#### 3.3.2. Radiomics Model 2

The most discriminating features, selected from clinical variables, LI-RADS qualitative imaging features, and quantitative imaging-based features by the hybrid descriptive-inferential feature extraction method, are reported in Supplementary Table S3. 

**Figure 5:**
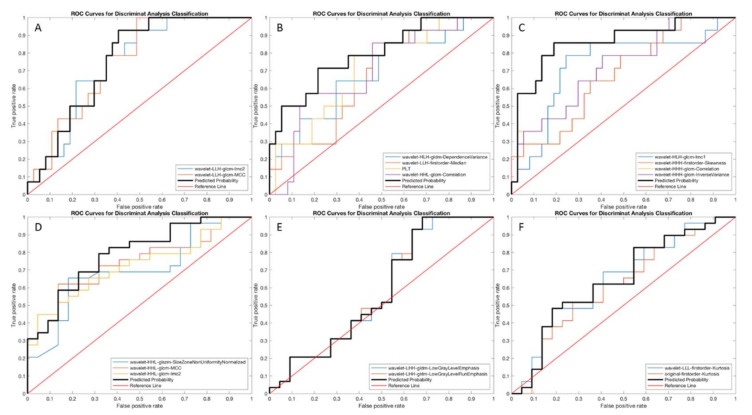
ROC curves of the Discriminant Analysis (radiomics model 2) for predicting complete response on portal-venous (**A**), 3′ transitional (**B**), and hepatobiliary (**C**) phases, and for the prediction of objective response on portal-venous (**D**), 3′ transitional (**E**), and hepatobiliary (**F**) phases.

Performances of the models tested by using the discriminant analysis are shown in Table 5. Corresponding ROC curves are illustrated in Figure 5. The model based on the HBP showed the highest diagnostic performance for predicting complete response, with an AUC of 0.861 (95% CI 0.737–0.984, *p* = 0.010), sensitivity of 75.5%, and specificity of 82.8%. Models on PVP and HBP demonstrated similar performances for predicting objective response, with an AUC of 0.791 (95% CI 0.667–0.915, *p* = 0.002), sensitivity of 71.3%, and specificity of 61.7% on PVP, and AUC of 0.790 (95% CI 0.649–0.931, *p* = 0.031), sensitivity of 58.8%, and specificity of 90.1% on HBP.

## 4. Discussion

Our study tested the performance of statistical and computational models based on the combination of clinical data, qualitative LI-RADS-defined imaging features, and radiomics features extracted from PVP, 3′ transitional phase, and HBP for the prediction of response after TAE in patients with unifocal HCC. Our results demonstrate that both models constructed through statistical and computational analyses had an almost perfect performance for the prediction of complete response using HBP images. For the prediction of objective response, the performances were higher on PVP and HBP when using the computational models, while performance of statistical-based models were lower in the test set. Notably, both models were mostly based on the selection of wavelet radiomics features, with most of the selected features being gray-level co-occurrence matrix. The differences in the final diagnostic performances may be related to the smaller number of patients included in the test set when testing the statistical models, especially when assessing complete response. Due to the large number of included features, the point-biserial correlation coefficient was adopted in the computational model to select the most discriminative features and to avoid overfitting problems. Importantly, only platelet count was significantly different between patients with complete response compared to non-responders, and none of the LI-RADS-based qualitative imaging features could predict response in our cohort. 

Nowadays, there are no robust predictors of optimal response after transarterial treatments in patients with HCC. Optimal patients’ selection for transarterial treatments is a key factor for prolonged survival outcomes [37]. Predictive models using only conventional clinical, biochemical, and radiological variables are not able to provide a reliable prediction of the complete radiological response in clinical practice [3,4]. This is concordant with our results, in which only platelet count was retained in the final combined models for the prediction of complete response. In clinical practice, indications for transarterial treatments are mostly based on HCC tumor stage, technical feasibility, and compensated hepatic function [37]. Nevertheless, intermediate stage HCC includes a wide spectrum of lesions with heterogeneous biological aggressiveness that cannot be anticipated by conventional radiological features. Radiomics has the potential to provide additional quantitative data that can be correlated with tumor biological aggressiveness [7]. Our study supports the applications of radiomics on gadoxetate disodium MRI for the assessment of patients before intraarterial treatments. The integration of radiomics features could be particularly relevant in clinical practice for selecting the optimal candidates to intraarterial treatments, while patients with radiomics features suggestive of poor response could benefit of early switching to alternative treatments, including the most recent systemic therapies with combination of immunotherapy and antiangiogenic drugs [38]. 

Few prior studies explored the value of MRI-based radiomics combined with clinical features for the prediction of tumor response after transarterial treatments [18,19,20,21,22]. Song et al. [18] built a nomogram for prediction of recurrence-free survival after TACE based on the combination of radiomics features of the entire tumor on PVP with extracellular contrast agent and clinical variables. Zhao et al. [21] presented a combined clinical-radiomics model for the prediction of objective response after TACE. In that study, the radiomics model on PVP on MRI with extracellular contrast agent showed the highest diagnostic performance (AUC of 0.830) in the validation cohort [21]. Kuang et al. [22] incorporated radiomics features on T2-weighted and arterial phase images in a nomogram to predict short-term response after TACE. Interestingly, similarly to our study, low platelet count was associated with poor response after treatment [22]. Our study proved that radiomics analysis may be used to predict response also on gadoxetate disodium-enhanced MRI. In our study the performance of final models including radiomics features was the highest on HBP images, with a sensitivity of 75.5% and specificity of 82.8% for the prediction of complete response, and a sensitivity of 58.8%, and specificity of 90.1% for the prediction of objective response, according to the computational analysis results and this is in line with prior literature demonstrating the role of HBP for HCC diagnosis [39,40,41]. Most importantly, no prior investigations compared the accuracy of radiomics-models against LI-RADS-based features. Interestingly, LI-RADS features seem to optimally predict recurrence-free survival and overall survival after curative surgical resection in patients with primary liver carcinomas, while there is scarce data on the predictive value of LI-RADS features in the context of locoregional treatments [42,43]. 

This single-center retrospective study has multiple limitations that need to be acknowledged. First of all, the study population was small, and the inclusion of only patients with single treatment-naïve HCC and available pre-treatment gadoxetate disodium-enhanced MRI acquired with the same protocol may have introduced selection biases. Further studies need to evaluate the performance of radiomics models in large multicentric cohorts before direct translation into clinical practice. Second, outcomes were based on response assessment using mRECIST criteria at one month, which is a moderate radiological surrogate of overall survival in patients undergoing transarterial treatments [44]. However, this remains the most widely used endpoint for treatment decisions. Third, not all the lesions were proven by pathology. The diagnosis of most HCCs treated in our study was made by categorization of observations as LR-5 using LI-RADSv2018, which means a 95% pooled proportion of lesions proven to be HCC at histopathological analysis [45]. Fourth, images were acquired using 1.5T MR scanners in our study; therefore, these results cannot be generalized to 3T MR scanners. We also did not evaluate T2-weighted sequences and diffusion weighted imaging, which were extensively investigated in prior studies [19,20]. Finally, HCC segmentation was performed manually. Although manual segmentation is still considered the reference standard for radiomics analysis, this is a time-consuming process that may be prone to inter-reader variability. 

## 5. Conclusions

Radiomics models based on gadoxetate disodium-enhanced MRI can achieve a good performance for the prediction of response in HCCs treated with TAE. The performance of radiomics-based models for predicting objective response is the highest when evaluating the portal venous phase and hepatobiliary phase images.

## Figures and Tables

**Table 1:** Differences in clinical characteristics according to the post-treatment response.

Characteristics	CR (*n* = 14)	PR + SD + PD (*n* = 37)	*p* Value	CR + PR (*n* = 29)	SD + PD (*n* = 22)	*p* Value
**Age** (years)	72.5 (65.0, 75.7)	74.0 (67.0, 78.5)	0.526	73.0 (65.0, 79.5)	73.5 (69.5, 77.2)	0.614
**Sex** Males Females	9 (64.3) 5 (35.7)	28 (75.7) 9 (24.3)	0.490	20 (69.0) 9 (31.0)	17 (77.3) 5 (22.7)	0.510
**Etiology of cirrhosis** Hepatitis C Hepatitis B NAFLD	12 (85.8) 1 (7.1) 1 (7.1)	31 (83.8) 5 (13.5) 1 (2.7)	0.649	24 (82.8) 4 (13.8) 1 (3.4)	19 (86.4) 2 (9.1) 1 (4.5)	0.864
**Albumin** (g/dL)	3.4 (3.3, 3.9)	3.6 (3.2, 3.9)	0.767	3.4 (3.2, 3.9)	3.7 (3.4, 4.0)	0.147
**Creatinine** (mg/dL)	0.8 (0.7, 0.9)	0.8 (0.7, 1.0)	0.670	0.8 (0.7, 1.0)	0.8 (0.7, 1.0)	0.803
**Bilirubin** (mg/dL)	0.8 (0.6, 1.1)	0.8 (0.6, 1.2)	0.983	0.8 (0.6, 1.4)	0.8 (0.6, 1.0)	0.661
**Platelet count** (×10^3^/μL)	115.1 (97.0, 170.7)	80.0 (59.5, 120.0)	**0.021**	101.0 (63.0, 130.5)	86.5 (60.2, 120.7)	0.697
**INR**	1.0 (1.0, 1.1)	1.0 (1.0, 1.2)	0.380	1.0 (1.0, 1.1)	1.0 (1.0, 1.2)	0.985
**α-fetoprotein** (ng/mL)	10.3 (3.7, 122.0)	5.0 (2.9, 47.1)	0.597	8.4 (2.7, 41.7)	5.0 (2.9, 121.2)	0.886
**Child-Pugh Class** A B	14 (100) 0 (0)	31 (83.8) 6 (16.2)	0.170	26 (89.7) 3 (10.3)	19 (86.4) 3 (13.6)	1.000
**Ascites**	0 (0)	8 (21.6)	0.088	5 (17.2)	3 (13.6)	1.000
**Varices**	10 (71.4)	29 (78.4)	0.715	22 (75.9)	17 (77.3)	0.906

Note. Continuous variables are expressed as median and interquartile range (25th to 75th percentile), categorical variables are expressed as numbers and percentages. Categorical variables were compared using the Pearson χ2 or Fisher exact test and continuous variables using the Mann–Whitney U test. Statistically significant values (*p* < 0.05) are highlighted in bold. Abbreviations: CR: Complete Response; PR: Partial Response; SD: Stable Disease; PD: Progressive Disease; NAFLD: Nonalcoholic Fatty Liver Disease.

**Table 2:** Differences in LI-RADS major and ancillary features favoring malignancy according to treatment response.

Characteristics	CR (*n* = 14)	PR + SD + PD (*n* = 37)	*p* Value	CR + PR (*n* = 29)	SD + PD (*n* = 22)	*p* Value
Size (mm)	23.0 (14.7, 27.0)	20.0 (16.0, 31.5)	0.642	22.0 (15.5, 28.0)	20.0 (16.0, 32.0)	0.593
Nonrim APHE	13 (92.9)	36 (97.3)	0.478	27 (93.1)	22 (100)	0.500
Nonperipheral “washout”	11 (78.6)	32 (86.5)	0.668	22 (75.9)	21 (95.5)	0.117
Enhancing “capsule”	6 (42.9)	17 (45.9)	0.843	12 (41.4)	11 (50.0)	0.581
Threshold growth	0 (0)	4 (10.8)	0.565	3 (10.3)	1 (4.5)	0.624
US visibility as discrete nodule	4 (28.6)	5 (13.5)	0.236	5 (17.2)	4 (18.2)	1.000
Subthreshold growth	5 (35.7)	9 (24.3)	0.490	6 (20.7)	8 (36.4)	0.214
Corona enhancement	1 (7.1)	2 (5.4)	1.000	2 (6.9)	1 (4.5)	1.000
Fat sparing in solid mass	1 (7.1)	3 (8.1)	1.000	3 (10.3)	1 (4.5)	0.625
Restricted diffusion	9 (64.3)	24 (64.9)	1.000	18 (62.1)	15 (68.2)	0.651
Mild-moderate T2 hyperintensity	7 (50.0)	20 (54.1)	0.796	13 (44.8)	14 (63.6)	0.183
Iron sparing in solid mass	0 (0)	0 (0)	NA	0 (0)	0 (0)	NA
Transitional phase hypointensity	9 (64.3)	33 (89.2)	0.093	21 (72.4)	21 (95.5)	0.060
HBP hypointensity	12 (85.7)	32 (86.5)	1.000	24 (82.8)	20 (90.9)	0.684
Nonenhancing “capsule”	0 (0)	1 (2.7)	1.000	1 (3.4)	0 (0)	1.000
Nodule-in-nodule architecture	1 (7.1)	3 (8.1)	1.000	2 (6.9)	2 (9.1)	1.000
Mosaic architecture	1 (7.1)	3 (8.1)	1.000	1 (3.4)	3 (13.6)	0.303
Fat in mass, more than adjacent liver	2 (14.3)	7 (18.9)	1.000	4 (13.8)	5 (22.7)	0.474
Blood products in mass	1 (7.1)	0 (0)	0.275	1 (3.4)	0 (0)	1.000

Note. Continuous variables are expressed as median and interquartile range (25th to 75th percentile), categorical variables are expressed as numbers and percentages. Categorical variables were compared using the Pearson χ^2^ or Fisher exact test and continuous variable (size) using the Mann–Whitney U test. NA: Not Available since this feature was never encountered. Statistically significant values (*p* < 0.05) are highlighted in bold. Abbreviations: CR: Complete Response; PR: Partial Response; SD: Stable Disease; PD: Progressive Disease; APHE: Arterial Phase Hyperenhancement; HBP: Hepatobiliary Phase.

**Table 3:** Performance of logistic model with ridge penalty (radiomics model 1) based on the selected features for predicting complete response and objective response (complete and partial response) in the training set.

	Sensitivity	Specificity	Accuracy	AUC (95% CI)	*p* Value
	**Prediction of complete response**				
**PVP**	100	100	100	1.000 (1.000–1.000)	<0.001
**3′ TP**	100	100	100	1.000 (1.000–1.000)	<0.001
**HBP**	100	90.0	92.5	0.984 (0.957–1.000)	<0.001
	**Prediction of objective response**				
**PVP**	87.0	64.7	77.5	0.872 (0.765–0.979)	<0.001
**3′ TP**	100	100	100	1.000 (1.000–1.000)	<0.001
**HBP**	94.1	100	97.5	1.000 (1.000–1.000)	<0.001

Note. Sensitivity, specificity, and accuracy are reported as percentages. The area under the receiver operating characteristic curve (AUC) with 95% confidence interval (CI) was calculated to assess the diagnostic performance. Abbreviations: PVP: Portal Venous Phase; 3′ TP: 3 min Transitional Phase; HBP: Hepatobiliary Phase.

**Table 4:** Performance of logistic model with ridge penalty (radiomics model 1) based on the selected features for predicting complete response and objective response (complete and partial response) in the test set.

	Sensitivity	Specificity	Accuracy	AUC (95% CI)	*p* Value
	**Prediction of complete response**				
**PVP**	87.5	33.3	72.7	0.667 (0.251–1.000)	0.431
**3′ TP**	75.0	33.3	63.6	0.750 (0.429–1.000)	0.127
**HBP**	100	100	100	1.000 (1.000–1.000)	<0.001
	**Prediction of objective response**				
**PVP**	100	40.0	72.7	0.733 (0.405–1.000)	0.163
**3′ TP**	40.0	66.7	54.5	0.667 (0.305–1.000)	0.367
**HBP**	20.0	100	63.6	0.600 (0.193–1.000)	0.630

Note. Sensitivity, specificity, and accuracy are reported as percentages. The area under the receiver operating characteristic curve (AUC) with 95% confidence interval (CI) was calculated to assess the diagnostic performance. Abbreviations: PVP: Portal Venous Phase; 3′ TP: 3 min Transitional Phase; HBP: Hepatobiliary Phase.

**Table 5:** Performance of Discriminant Analysis (radiomics model 2) based on the selected features for predicting complete response and objective response (complete and partial response).

	Sensitivity	Specificity	Accuracy	AUC (95% CI)	*p* Value
	**Prediction of complete response**				
**PVP**	66.6	56.6	63.8	0.757 (0.626–0.888)	0.002
**3′ TP**	66.1	72.8	67.9	0.795 (0.654–0.936)	0.024
**HBP**	75.5	82.8	77.5	0.861 (0.737–0.984)	0.010
	**Prediction of objective response**				
**PVP**	71.3	61.7	65.8	0.791 (0.667–0.915)	0.002
**3′ TP**	54.1	65.6	60.7	0.585 (0.414–0.755)	0.049
**HBP**	58.8	90.1	76.7	0.790 (0.649–0.931)	0.031

Note. Sensitivity, specificity, and accuracy are reported as percentages. The area under the receiver operating characteristic curve (AUC) with 95% confidence interval (CI) was calculated to assess the diagnostic performance. Abbreviations: PVP: Portal Venous Phase; 3′ TP: 3 min Transitional Phase; HBP: Hepatobiliary Phase.

## Data Availability

Data will be made available by corresponding authors upon reasonable request.

## Supplementary Materials

The following supporting information can be downloaded at: https://www.mdpi.com/article/10.3390/diagnostics12061308/s1, Supplementary Table S1: Acquisition parameters of post-contrast axial T1-weighted three-dimensional gradient-recalled echo sequences with fat suppression at 1.5T MRI scanners; Supplementary Table S2: Most discriminant features identified by using a first features selection performed identified by the statistical model by a logistic regression with elastic net penalty (radiomics model 1), while the final model was a logistic model with ridge penalty on the selected features in order to find an uncorrelated solution of clinical variables for reduction and selection of clinical variables, LI-RADS qualitative features, and radiomics features according to the post-treatment response; Supplementary Table S3: Most discriminant features identified by the computational system based on point-biserial-correlation coefficient (radiomics model 2) for reduction and selection of clinical variables, LI-RADS qualitative features, and radiomics features according to post-treatment response.
