# Supplementary material for: Radiomics Analysis on Gadoxetate Disodium-Enhanced MRI Predicts Response to Transarterial Embolization in Patients with HCC
Source: Diagnostics (Basel). 2022 May 24;12(6):1308. doi: 10.3390/diagnostics12061308 (PMC9221802; doi:10.3390/diagnostics12061308)
Supplement: Supplementary file 1 [file diagnostics-12-01308-s001.zip › diagnostics-1685612-supplementary.pdf]

**Parameters**	**Signa Excite, GE Healthcare**	**Achieva, Philips Healthcare**
Repetition Time (ms)	3.8	4.4
Echo Time (ms)	1.8	2.1
Flip Angle (degrees)	12	10
Number of slices	92–116	83–110
Slice Thickness (mm)	4.4	5
Reconstruction Interval (mm)	2.2	2.3
Acquisition Matrix	256 x 256	270 x 188
Field of View (mm)	350–400 x 350–400	350–400 x 350–400
Number of Excitations	0.71	1

Supplementary Table S1: Acquisition parameters of post-contrast axial T1-weighted three-dimensional gradient-recalled echo sequences with fat suppression at 1.5T MRI scanners.

	Prediction of complete response		Prediction of objective response	
	Feature	*p* value	Feature	*p* value
**PVP**	original_glszm_SmallAreaEmphasis	0.017	wavelet-HHL_glszm_SizeZoneNonUniformityNormalized	0.049
	wavelet.LHH_firstorder_Skewness	0.011		
**3' TP**	Platelet count	0.023	wavelet.HLL_glcm_ClusterShade	0.027
	TP hypointensity	0.030	wavelet.LHH_glszm_SmallAreaEmphasis	0.044
	wavelet.LLH_ngtdm_Strength	0.038	wavelet.LLH_firstorder_Mean	0.020
**HBP**	Platelet count	0.034	TP hypointensity	0.034
	wavelet.HHH_firstorder_Skewness	0.027	original_glszm_LargeAreaHighGrayLevelEmphasis	0.044
	wavelet.LLH_glcm_Correlation	0.031	wavelet.HHL_glcm_Idmn	0.041
			wavelet.HHL_ngtdm_Contrast	0.022
			wavelet.LHL_glcm_Imc1	0.012
			wavelet.LHL_glcm_Imc2	0.014
			wavelet.LHL_gldm_LargeDependenceLowGrayLevelEmphasis	0.049

Supplementary Table S2: Most discriminant features identified by using a first features selection performed identified by the statistical model by a logistic regression with elastic net penalty (radiomics model 1), while the final model was a logistic model with ridge penalty on the selected features in order to find an uncorrelated solution of clinical variables for reduction and selection of clinical variables, LI-RADS qualitative features, and radiomics features according to the post-treatment response. **Abbreviations**: PVP: Portal Venous Phase; 3' TP: 3 minutes Transitional Phase; HBP: Hepatobiliary Phase.

	Prediction of complete response		Prediction of objective response	
	Feature	*p* value	Feature	*p* value
**PVP**	wavelet-LLH_glcm_Imc2	0.002	wavelet-HHL_glszm_SizeZoneNonUniformityNormalized	< 0.001
	wavelet-LLH_glcm_MCC	0.005	wavelet-HHL_glcm_MCC	< 0.001
			wavelet-HHL_glcm_Imc2	< 0.001
**3' TP**	wavelet-HLH_gldm_DependenceVariance	0.009	wavelet-LHH_gldm_LowGrayLevelEmphasis	0.009
	wavelet-LLH_firstorder_Median	0.005	wavelet-LHH_glrlm_LowGrayLevelRunEmphasis	0.030
	Platelet count	< 0.001		
	wavelet-HHL_glcm_Correlation	0.001		
**HBP**	wavelet-HLH_glcm_Imc1	0.002	wavelet-LLL_firstorder_Kurtosis	0.021
	wavelet-HHH_firstorder_Skewness	< 0.001	original_firstorder_Kurtosis	0.041
	wavelet-HHH_glcm_Correlation	< 0.001		
	wavelet-HHH_glcm_InverseVariance	< 0.001		

Supplementary Table S3: Most discriminant features identified by the computational system based on point-biserial-correlation coefficient (radiomics model 2) for reduction and selection of clinical variables, LI-RADS qualitative features, and radiomics features according to post-treatment response. **Abbreviations**: PVP: Portal Venous Phase; 3' TP: 3 minutes Transitional Phase; HBP: Hepatobiliary Phase.
